# Semax, a Copper Chelator Peptide, Decreases the Cu(II)-Catalyzed ROS Production and Cytotoxicity of aβ by Metal Ion Stripping and Redox Silencing

**DOI:** 10.1155/bca/4226220

**Published:** 2025-06-03

**Authors:** Marianna Flora Tomasello, Maria Carmela Di Rosa, Irina Naletova, Michele Francesco Maria Sciacca, Alessandro Giuffrida, Giuseppe Maccarrone, Francesco Attanasio

**Affiliations:** ^1^Institute of Crystallography, CNR, P. Gaifami 18, Catania 95126, Italy; ^2^Department of Chemical Sciences, University of Catania, A. Doria 6, Catania 95125, Italy

**Keywords:** amyloid-β, bioinorganic chemistry, copper, oxidative stress, ROS, Semax

## Abstract

Alzheimer's disease (AD) is the most common neurodegenerative disorder associated with cognitive decline and loss of memory. It is postulated that the generation of reactive oxygen species (ROS) in Fenton-like reaction connected with Cu(II)/Cu(I) redox cycling of the Cu(II)-aβ complex can play a key role in the molecular mechanism of neurotoxicity in AD. Semax (Met-Glu-His-Phe-Pro-Gly-Pro) is a synthetic regulatory peptide that possesses a high affinity for Cu(II) ions. The ability of the peptide Semax to inhibit the copper-catalyzed oxidation of aβ was studied in vitro and discussed. The results indicate that Semax is able to extract Cu(II) from Cu(II)-aβ species as well as to influence the redox cycling of the Cu(II)-aβ complex and decrease the level of associated ROS production. Finally, our data suggest that Semax shows cytoprotective properties for SH-SY5Y cells against oxidative stress induced by copper-catalyzed oxidation of the aβ peptide. This study provides valuable insights into the potential role of Semax in neurodegenerative disorders and into the design of new compounds with therapeutic potential for AD.

## 1. Introduction

Alzheimer's disease (AD) is a neurodegenerative disorder, a progressive and fatal brain disease characterized by the presence of intracellular neurofibrillary tangles and extracellular deposition of amyloid-β (aβ) peptide in the form of senile plaques [1–6]. Clinically, AD is characterized by a progressive loss of memory, a degeneration of cognitive skills [7, 8], and a loss of neurons that occurs in the cerebral cortex and hippocampus of the brain [9]. Furthermore, AD is triggered by age and originates in specific parts of the brain with elevated levels of metal ions. Among the main and consistent features of AD, oxidative stress and imbalances in the metal ions homeostasis have a critical role [10, 11]. Several studies have shown that the AD-affected brain contains elevated concentrations of transition metals accumulated in amyloid plaques, mainly copper, zinc, and iron, suggesting that these metal ions actively take part in the etiology of the pathology by interacting with aβ peptide [3, 9, 12]. These essential transition metal ions have important functions in the brain [13] and maintaining their homeostasis is critical for the brain functions. Abundant research has pointed out the deleterious roles of metal ions in the development of AD. These include (i) the aggregation of aβ peptides to form senile plaques and neurofibrillary tangles [14–16] and/or (ii) the increase of metal ion–mediated oxidative stress [9, 17]. Copper metabolism is closely regulated and this ion is normally bound to proteins [18, 19], but the so-called Cu pool loosely bound can catalyze the production of reactive oxygen species (ROS) [20]. In particular, due to its redox-active nature (Cu^2+^/Cu^+^), copper reacts with molecular oxygen (O_2_) and generates ROS such as superoxide, hydrogen peroxide, and hydroxyl radicals [21]. This ROS overproduction could damage biomolecules or overwhelm antioxidant mechanisms leading to an increased oxidative stress and inflammation.

The redox cycling of copper requires its reduction by biological components, including ascorbate, which is actively present in the brain at high concentrations [22]. Increasing evidence suggests that the neuronal cell lost in AD is linked, at least partially, to an excessive free radical generation [23], and the noncontrolled redox-active metal ions, especially copper, in the brain of patients with AD should be considered at the root of a cascade of events causing the intense oxidative damage in the AD brain. One of the proposed mechanisms to explain the aβ toxicity includes the production of ROS by the aberrant binding of redox active copper ions to aβ [24]. In order to elucidate copper-mediated events in AD pathogenesis, the redox activity and the coordination chemistry of Cu(II)-aβ have been the focus of several studies [25–30]. Indeed, the binding of aβ to redox active metals (i.e., Cu) can facilitate redox cycling leading to the production of ROS and increasing the oxidative stress [30–37]. In vitro studies have shown that, in the presence of copper, aβ can produce H_2_O_2_ by reducing molecular oxygen [38–40] and catalyzing ascorbate oxidation with the generation of hydroxyl radical (•OH) through Fenton reaction [41–44]. Interestingly, recent evidence showed that Cu(II) enhances the effect of aβ on microglial activation and neurotoxicity involving mitochondrial ROS production [45] and potentiates the spatial memory deficit induced by aβ in the hippocampus of rats [46]. Based on the hypothesis that a dis-homeostasis of copper ions in the AD might contribute to pathological situations in aging brains, copper chelation and its redox-silencing may represent a critical event in preventing the progression of neurodegenerative diseases. For this purpose, copper ion chelators have been widely studied and proposed as a potential strategy for AD therapy to attenuate abnormal metal-protein interactions that lead to increased free radical toxicity [47–53]. In particular, N4-tetradentate copper specific ligands have been taken into consideration for their ability to strongly bind copper without interfering with zinc homeostasis, the most common metal ion involved in neuronal signal transduction [54–58].

Semax (Met-Glu-His-Phe-Pro-Gly-Pro) is a synthetic peptide based on regulatory peptides (ACTH) and in particular on the ACTH(4–10) sequence. This fragment is practically devoid of hormonal effects but completely preserves the neurotrophic activity of the entire molecule. Semax consists of the ACTH(4–7) fragment and the C-terminal tripeptide Pro-Gly-Pro (PGP) [59], has no hormonal effects, and preserves the entire behavioral effects of its precursor with an increased stability to the proteolysis in tissues and body fluids. Indeed, it has been shown that the addition of the sequence enriched in proline residues to the C-terminus of ACTH(4–7) results in an increased duration of the behavioral effects of the peptide [60–62]. Semax affects several biological processes involved in the function of various systems and exhibits neuroprotective, neurotrophic, and nootropric properties, stimulates learning and memory formation in rodents and humans [63–66], and could represent the basis for drugs to be used for the treatment of CNS diseases [61]. Despite having a wide range of biological activity, the molecular mechanisms underlying the action of Semax remain unclear. A large-scale study of the action mechanism at the transcriptome level was of particular interest [65, 67–70].

Recently, we demonstrated that (i) Semax possesses a high affinity for Cu(II) ions (ATCUN binding site, conditional K_D_ 1.3 10^−15^ M at pH = 7.4) [71] with respect to Zn(II) ions (K_D_ 1.8 × 10^− 5^ M at pH = 7.4) [72] and a protective ability against metal-induced cell toxicity [71]; (ii) acetylation of the N-terminal amino group affects copper(II) and zinc(II) chelation properties of Semax and the inhibition of the Cu(II)-mediated ascorbic acid oxidation [72]; and (iii) Semax affects copper-induced aβ aggregation and amyloid formation in artificial membrane models [73].

Here we report the inhibition of copper-catalyzed oxidation of aβ by Semax. We employed aβ_1–16_ and aβ_1–28_ as well-established model peptides for our in vitro studies since they contain all the copper binding residues and do not readily precipitate or aggregate in the experimental condition used. Aβ_1–40_ was used either for in vitro studies or for in-cell experiments. We tested in vitro the influence of Semax on the Cu(II) ion interaction with aβ peptides and on the ROS production in the presence of L-ascorbic acid (ASC) by ultraviolet (UV)-absorbance measurements and coumarin-3-carboxylic acid (3-CCA) fluorescence assay. Finally, the inhibitory effect of Semax on ROS generation and cytotoxicity of Cu(II)-aβ complex were also estimated on human neuroblastoma SH-SY5Y cells line by using flow cytometry and MTT assay.

## 2. Results and Discussion

### 2.1. Aβ-Cu^2+^–Induced ROS

It is hypothesized that ROS contribute to the neuronal failure in AD brains. Aβ plays a key role in the metal ion–mediated ROS production during oxidative stress [24, 74–76]. Physiologically, ROS and other free radical species are eliminated by “sacrificial scavengers,” which convert radicals by reduction reactions. Ascorbate is a physiological reducing agent present at a high concentration, in particular in the brain (up to 10 mM in neurons) [22]. It can convert radicals into nonradicals, for instance HO^•^ to HO^−^, or O_2_^−^ to H_2_O_2_. Transferring an electron to a nonradical species can also result in reactions that lead to the formation of radicals; however, these reactions are normally not efficient [77], unless they are catalyzed [78]. Cu(II) and Cu(II) complexes (including aβ complexes) are known to catalyze the production of ROS in the presence of oxygen and ascorbate [44, 79–81] through a Fenton–Haber–Weiss reaction [78].

The rate of ascorbate consumption, as one of the reactants of the Fenton–Haber–Weiss reaction, can provide an indirect index of the Cu(II) species' ability to generate ROS [48]. Moreover, the formation of the very reactive hydroxyl radical (OH^•^) can be monitored by measuring the fluorescence of 7-hydroxycoumarin-3-carboxylate (7-OH-CCA) which is the product of the oxidation of 3-CCA.

**Figure 1:**
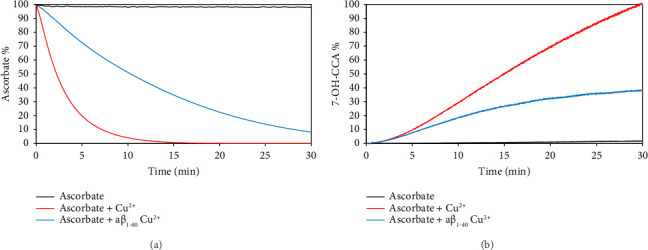
(a) Ascorbate (150 μM) consumption when alone (black curve), in presence of 20 μM Cu^2+^ (red curve), and in presence of both 20 μM aβ_1–40_ and 20 μM Cu^2+^ (blue curve). (b) Formation of OH^•^ measured by fluorescence of 7-OH-CCA formed in presence of 150 μM ascorbate alone (black curve), in presence of 150 μM ascorbate + 20 μM Cu^2+^ (red curve), and in presence of 150 μM ascorbate + 20 μM aβ_1–40_ + 20 μM Cu^2+^ (blue curve). All measurements were performed in 10 mM phosphate buffer, pH = 7.4.

**Figure 2:**
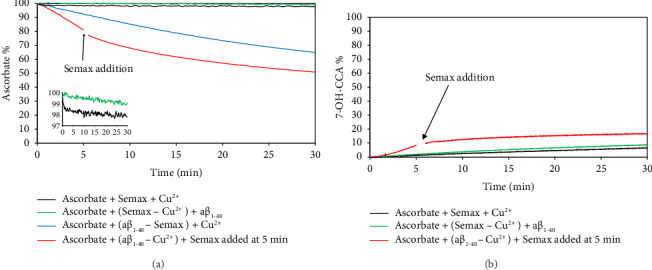
(a) Ascorbate (150 μM) consumption in presence of 25 μM Semax + 20 μM Cu^2+^ (black curve), in presence of the preformed 20 μM complex (Semax − Cu^2+^) + 20 μM aβ_1–40_ (green curve), in presence of 20 μM (aβ_1–40_ − Semax) + 20 μM Cu^2+^ (blue curve), and in presence of 25 μM Semax added after 5 min at the preformed complex 20 μM (aβ_1–40_ − Cu^2+^). (b) Formation of OH^•^ measured by fluorescence of 7-OH-CCA formed in presence of 150 μM ascorbate alone (black curve), in presence of the preformed 20 μM complex (Semax − Cu^2+^) + 20 μM aβ_1–40_ (green curve), and in presence of 25 μM Semax added after 5 min at the preformed complex 20 μM (aβ_1–40_ − Cu^2+^). All measurements were performed in 10 mM phosphate buffer, pH = 7.4.

In absence of any metal ion, ascorbate solution is stable over time (Figures 1(a) and 1(b), black curve). As expected, the addition of free Cu(II) catalyzes the complete ascorbate consumption (Figure 1(a), red curve) and HO^•^ production as evidenced by the increase in the fluorescence of 7-OH-CCA (Figure 1(b), red curve). Indeed, after 30 min, we observed the total ascorbate consumption (Figure 1(a), red curve) and the conversion of 3-CCA into the fluorescent species 7-OH-CCA (Figure 1(b), red curve) [43, 78, 82]. The presence of aβ in the ascorbate solution produced a reduction in both the rate and the level of ascorbate consumption after the addition of Cu(II) (Figure 1(a), blue curve) and HO^•^ production (Figure 2(b), blue curve). Notably, aβ_1–40_ does not show any difference in the distribution of the concentration of monomeric and oligomeric species over the time length of the experiment, as observed by western blot (Figure S1). We did not observe any significant difference in ascorbate consumption and generation of HO^•^ for aβ_1–16_, aβ_1–28_ (aβ fragments which are known to form complexes with Cu(II) [83–85]) and aβ_1–40_ (Figure S2), and thus the decrease in the efficiency of the Fenton–Haber–Weiss reaction is due, as expected, only to the aβ:Cu(II) complex formation. In presence of aβ:Cu(II) complexes, we observed a significant difference between ascorbate consumption (∼100% as observed in the experiment with “free” copper ion) at the end of the reactions and the amount of HO^•^ (significantly lower than that observed in the presence of “free” copper ion). This behavior is in agreement with literature data which report a moderate ROS generation of aβ:Cu(II) complex compared to “free” copper, due to a more difficult redox-cycling [35, 79]. Moreover, the results are in agreement with the observation that aβ scavenges the HO^•^ due to its proximity to the site of ROS production and is oxidized regardless of ascorbate concentrations [78].

### 2.2. Semax Inhibits OH^•^ Generation and Ascorbate Consumption Induced by Cu^2+^ and aβ-Cu^2+^

Semax is a peptide with ATCUN motif having [CuH_−2_L]^2−^ as the only very stable complex species able to strongly chelate copper ions at physiological pH and a very negative formal redox potential [71].

In a previous work, we showed that the Cu(II)-Semax complex species are unable to catalyze the ascorbic acid oxidation [72]. Here we tested the ability of Semax peptide to inhibit the Cu(II)-induced ROS production of aβ peptides and the relative ascorbate consumption. We used an excess of peptide (1.2:1 peptides/Cu(II) molar ratio) to avoid the presence of free Cu(II) in solution.

In the presence of Semax, the copper redox cycling was completely silenced. The experiment was carried out by adding copper to a solution containing ascorbate and Semax. We observed an immediate consumption of a very small amount of ascorbate (Figure 2(a)), followed by the complete inhibition of Cu(II)-mediated ascorbic acid oxidation (Figure 2(a), black curve). The fast initial ascorbate consumption is probably due to the fast kinetic Cu(II)/Cu(I) redox reaction preceding the formation of the thermodynamically stable Semax:Cu(II) complex. The trend of the HO^•^ species production reflected the ascorbate consumption (Figure 2(b), black curve). According to the Fenton–Haber–Weiss reaction mechanism, the ability of Semax to strongly chelate Cu(II) prevents accessibility of oxygen and ascorbate to the metal ions, hindering the electron transfer process from ascorbate to Cu(II) ions and, in turn, the reoxidation by oxygen generating ROS.

Furthermore, after a successive addition of equimolar amount of aβ peptide to the sample with preformed Semax:Cu(II) complex, we did not observe ascorbate consumption (Figure 2(a), green curve) nor formation of HO^∙•^ species (Figure 2(b), green curve), and thus once the Cu(II) is bound to Semax, it cannot be coordinated by aβ peptide. This is not surprising due to the very low value of the conditional dissociation constant of the complex (^c^K_d_ = 1.3 × 10^−15^ M). The ability of Semax to form a stable Cu(II) complex in the presence of aβ peptide as well as to extract copper by aβ has been investigated also by UV-visible (UV-Vis) spectra as reported in Figure S3: when 1 mol equivalent of Semax was added to aβ:Cu(II) complex (absorbance spectrum band at 620 nm), the absorbance spectrum exhibited a band shift at 522 nm, specific for the formation of 4N square planar geometry Semax:Cu(II) complex [71]. This reaction was not reversible: when aβ was added into a Semax:Cu(II) complex solution, no change in the absorbance spectrum was observed, indicating that copper remained chelated to Semax. The result is consistent with the higher Semax affinity for copper with respect to aβ by several orders of magnitude [71, 86].

Furthermore, we performed a series of experiments to evaluate if in the presence of ascorbic acid: (i) Semax competes with aβ for the formation of Cu(II) complex in solution and (ii) Semax is able to extract copper ion from preformed aβ:Cu(II) complex.

Initially, we measured the consumption of ascorbate induced by the addition of Cu(II) to an ascorbate solution containing an equimolar amount of aβ and Semax. Interestingly, we observed a small and slow ascorbate consumption (Figure 2(a), blue curve). Afterward, Cu(II) was added to a solution containing ascorbate and aβ peptides, and both ascorbate consumption (Figure 2(a), red curve) and HO^•^ production (Figure 2(b), red curve) were monitored. After 5 min of reaction, an equimolar amount of Semax was added to the solution (indicated by an arrow in Figures 2(a) and 2(b), respectively). We observed a change in the rate as well as in the level of ascorbate consumption and HO^•^ production, suggesting that Semax is able to extract the metal ion from preformed aβ:Cu(II) complex. The same behavior was observed for aβ_1–16_ and aβ_1–28_ (Figures S4 and S5, respectively). In the last two experiments (Figure 2), we expected the complete silencing of the Cu(II) redox cycle due to the very high stability of Semax:Cu(II) complex. What we observed was a small, but significant, consumption of ascorbate and production of OH^•^; thus, even if Semax is able to form redox inert Cu(II) complexes and to extract Cu(II) from aβ:Cu(II) complex, the Fenton–Haber–Weiss reaction was not completely inhibited (Figures 2(a) and 2(b), black curves). This behavior, showing that redox inert Cu(II) ATCUN motif is not enough to inhibit ROS production, has been previously shown and explained by other authors [57, 58].

We hypothesized that, due to the starting conditions, the equilibrium between kinetic/thermodynamic competitive processes is the driven force determining the fate of the reactions. Thus, after addition, in the presence of ascorbic acid, Semax, and aβ, a small amount of Cu(II) undergoes reduction to Cu(I) probably because copper reduction process is kinetically favored with respect to the Semax-Cu(II) complex formation. The resulting Cu(I) cannot form complex with Semax but could form complex with aβ, which is known to form relatively weak complex with Cu(I) [86–88]. Thus, Cu(I) continues to be available to be oxidized and the redox cycle slowly proceeds, with a moderate ROS production.

### 2.3. Influence of Ligand-Cu(I) Affinity on Semax-Cu(II) Reactivity

**Figure 3:**
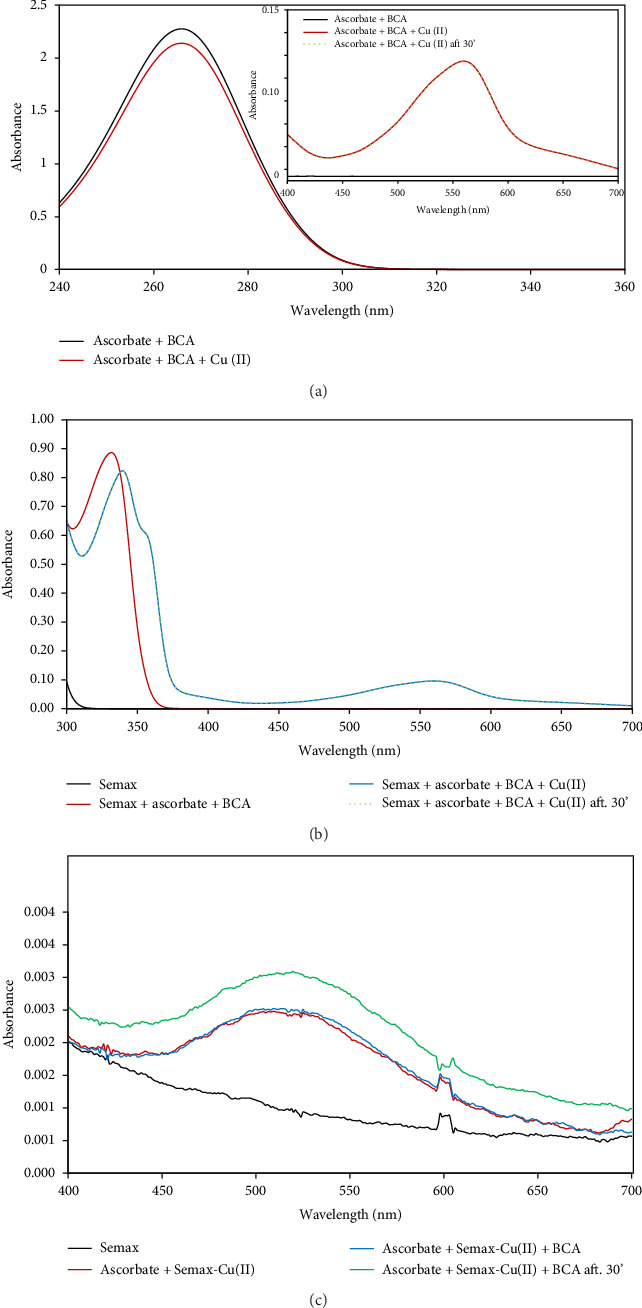
(a) UV spectra of 150 μM ascorbate + 52 μM BCA solution (black curve) and 150 μM ascorbate + 52 μM BCA after addition of 20 μM Cu(II) (red curve). The BCA contribution is subtracted by the reported UV spectra. Inset: Vis spectra of the Cu(I)-(BCA)_2_ complex formation immediately after Cu(II) addition (red curve) and after 30 min (blue dot). (b) UV-Vis spectra of 25 μM Semax (black curve), 25 μM Semax + 150 μM ascorbate + 52 μM BCA solution (red curve), 25 μM Semax + 150 μM ascorbate + 52 μM BCA solution immediately after addition of 20 μM Cu(II) (blue curve), and 25 μM Semax + 150 μM ascorbate + 52 μM BCA solution after 30 min from the addition of 20 μM Cu(II) (green dot). (c) Vis spectra of 150 μM Semax (black curve), 150 μM ascorbate + 20 μM Semax-Cu(II) (red curve), 150 μM ascorbate + 20 μM Semax-Cu(II) + 52 μM BCA (blue curve), and 150 μM ascorbate + 20 μM Semax-Cu(II) after 30 min from the addition of 52 μM BCA (green curve). All measurements were performed in 10 mM phosphate buffer, pH = 7.4.

To verify our hypothesis, we performed experiments in the presence of bicinchoninic acid (BCA) which is known to be an efficient ligand for Cu(I) ions. Two series of UV-Vis measurements were carried out (Figure 3).

The first experiment was carried out adding Cu(II) to a solution containing ascorbic acid and BCA (Figure 3(a)) and the spectrum was recorder after 1 min. The data showed a decrease of ascorbic acid spectrum intensity (Figure 3(a)) and the simultaneous appearance of a band with a *L*_max_ at 560 nm related to the formation of the Cu(I) (BCA)_2_ complex (Figure 3(a), inset, red curve). After 30 min, no change in the spectrum intensity was observed (Figure 3(a), inset, dot blue curve).

Indeed, the simultaneous presence of ascorbic acid and BCA leads to a fast and quantitative reduction of the copper(II) and to the formation of the corresponding Cu(I) (BCA)_2_ complex.

Taking into consideration the molar extinction coefficient value of 6.6 × 10^3^ mol^−1^·cm^−1^ [89] and the abs value (0.105) at *L*_max_, we found, from Lambert–Beer law, a concentration value for the copper(I) (BCA)_2_ complex equivalent to the value of Cu(II) added (16 µM).

The second experiment was carried out adding Cu(II) to a solution containing ascorbic acid, BCA, and Semax (Figure 3(b)). Even in the presence of this strong Cu(II) ion chelator, the data show that Cu(II) undergoes a reduction reaction with a formation of the Cu(I) (BCA)_2_ complex. On the contrary, if the complex Cu(II) Semax is already formed, the reduction did not occur (Figure 3(c), red curve) and after adding of BCA, no Cu(I)-(BCA)_2_ complex is present (blue and green curve). This behavior is easily understandable taking into account the equilibria involved (see supporting information, S6).

Hence, the complex Cu(I) (BCA)_2_ (log *β* = 38.64) is much more stable than Cu(II)-Semax (log *β* = 27.66) [71], and thus, in the presence of BCA, Cu(II) is readily reduced to Cu(I). On the other hand, if the complex Cu(II)-Semax is already formed, the decrease of concentration of ascorbic acid was negligible, indicating that ascorbate cannot reduce the Cu(II), even in the presence of BCA that should give rise to the more stable complex. This behavior is probably due to different rates of reaction (kinetics of redox and thermodynamics of complex formation).

### 2.4. Semax Prevents Cu(II)-Ascorbate and Cu(II) Ascorbate-aβ–Induced ROS Generation in Cultured Neuroblastoma Cells

Free radicals are generally acknowledged as serious threat for the correct cell functioning [90, 91]. Notably, oxidative stress significantly contributes to the development of aβ toxicity [92, 93]. Based on these findings, we investigated intracellular ROS production in SH-SY5Y cells by introducing either Cu(II) + ascorbate or Cu(II) + ascorbate + aβ into the extracellular medium. ROS were determined by reading the dichlorofluorescein diacetate (H2DCFDA) staining in flow cytometry, as described in the experimental section.

**Figure 4:**
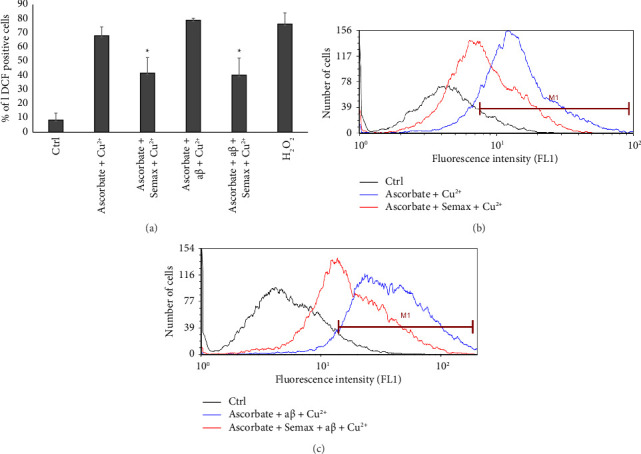
(a) Cells were treated as discussed in the experimental section and ROS monitored by measuring the DCF fluorescence by flow cytometry. Conditions were: 20 μM Cu(II), 300 μM ascorbate, 25 μM aβ, 25 µM Semax, and 100 μM H_2_O_2_ as a positive control. Histograms are representative for three sets of independent experiments, each performed in triplicate and based on 20,000 events for each sample. Bars: mean ± SEM; ∗ indicates a significant difference (*p* < 0.001, *χ*^2^ test). (b, c) Representative curves showing the distribution of SH-SY5Y population based on the intensity of DCF signal. Semax clearly lowers the fluorescence increase induced by either Cu(II)-ascorbate and Cu(II) ascorbate-aβ. The M1 gate (region) was chosen considering the fluorescence intensity shift between untreated cells and cells exposed to 100 μM H_2_O_2_.

Approximately 10% of untreated cells, which represent the negative control, show significant 2′,7′-dichlorofluorescein (DCF) signal, the end product of the oxidation of H2DCFDA, beyond the threshold established (M1 region). Exposure to 100 μM H_2_O_2,_ used as a positive control, leads to roughly 75% of cell population with increased DCF fluorescence based on the M1 region considered to this purpose (Figure 4(a)). Upon treatments with either Cu(II) + ascorbate (Figure 4(b), blue curve) or with Cu(II) + ascorbate + aβ (Figure 4(c), blue curve), the percentage of DCF positive cells (M1 region) resulted, respectively, in 68% and 79% (Figure 4(a)) of the population. These results suggest an increase in free oxygen radicals produced through the Fenton–Haber–Weiss reaction within SH-SY5Y cells. In particular, we observed that the presence of aβ significantly increases the ROS levels produced by Cu^2+^ reduction cycle. The presence of stoichiometric amounts of Semax significantly decreases the percentage of DCF positive cells, both in absence (Figure 4(b), red curve) or in presence of aβ (Figure 4(c), red curve) with a percentage of 41% and 40% of cells in the M1 region, respectively. These results are in agreement with the in vitro measurements shown above and suggest that Semax can prevent ROS-induced SH-SY5Y cell death by inhibiting Cu(II) + ascorbate and Cu(II) + ascorbate + aβ, which catalyze ROS generation.

### 2.5. Semax Prevents Cu(II)-Ascorbate and Cu(II) Ascorbate-aβ–Induced Cell Death in Cultured Neuroblastoma Cells

Considering that both ROS production and aβ oligomerization are harmful to neuronal cells, we measured by the MTT assay the viability of neuroblastoma SH-SY5Y cells exposed to either Cu(II) + ascorbate or Cu(II) + ascorbate + aβ and the effect of metal chelation by Semax in preventing cell death.

**Figure 5:**
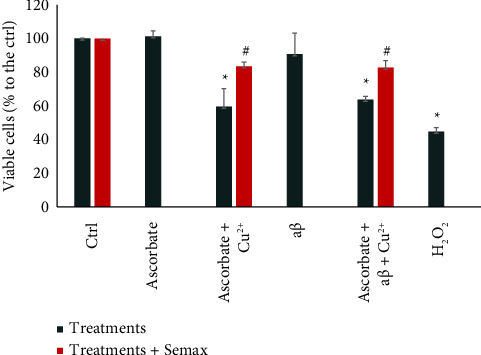
Cell viability was measured by MTT assay. SH-SY5Y cells were seeded and treated for 24 h with 20 μM copper/300 μM ascorbate or 20 μM copper/300 μM/20 µM aβ in the absence (black series) or presence (red series) of 25 μM Semax. Changes in the reductase activity are expressed as percentage to the control made using peptide-free vehicle (PBS). Values represent the mean ± SEM of three independent experiments performed in triplicate. ∗ indicates significance at *p* < 0.05 vs. Ctrl. # indicates significance of Semax reversion vs. the value produced by the same respective treatment in absence of Semax (one-way ANOVA + Tukey's multiple comparison test). The inset shows the MTT values for the negative controls (300 μM ascorbate alone, and 20 μM aβ: no cytotoxicity reported in this conditions) and the positive control (100 μM H_2_O_2:_ ROS-induced cell death).

Viability of SH-SY5Y cells exposed to aβ monomer is similar to that observed for nontreated cells (Figure 5). These results are consistent with the observation that aβ monomer is nontoxic to cells as reported in the literature [94, 95].

Exposure to Cu(II) + ascorbate and Cu(II) + ascorbate + aβ reduced the cells' viability to 59% and 63%, respectively, compared to the control. This result, compared with that obtained with aβ monomer alone, underlines the importance of the cytotoxic potential of Cu(II) + ascorbate + aβ strictly linked to ROS production.

As expected, the presence of Semax significantly lowered the cytotoxic effect of Cu(II) + ascorbate and Cu(II) + ascorbate + aβ (viability of 83% and 82%, respectively), which is consistent with all the other data showed. 100 μM H_2_O_2_ was used as positive control for ROS-induced cell death.

## 3. Conclusion

In conclusion, this study demonstrated that Semax, through metal ion stripping and redox silencing, is able to reduce both Cu(II)-catalyzed ROS production and the consequent aβ cytotoxicity. We showed that Semax-Cu(II) complex is highly stable and resistant to the ascorbate reduction, and Semax is able to extract Cu(II) from aβ peptide. However, the data show that the presence of the ACTUN motif with a high affinity constant (^c^K_d_ = 1.3 10–15 M) does not guarantee the complete copper redox silencing if Cu(I) chelator is present in solution. The driving force of this phenomenon can be attributed to the dynamic equilibrium between the kinetic copper reduction process and the thermodynamics of Semax-Cu(II) and/or aβ-Cu(I) complex formation. These observations are in accord with the behavior previously reported for other ACTUN motifs by various authors [57, 96–98].

Moreover, we show that Semax prevents Cu(II) + ascorbate and Cu(II) + ascorbate + aβ–induced cell death and ROS generation in cultured neuroblastoma cells.

Based on our previous study demonstrating Semax's ability to counteract copper-induced aβ aggregation and amyloid formation in artificial membrane models [73] and our findings on Semax's significant reduction of ROS production by the aβ-Cu(II) complex, along with its known roles in various biological processes [61, 63–66], Semax could be considered a potential adjuvant in therapeutic approaches for neurodegenerative diseases such as AD.

## 4. Materials and Methods

### 4.1. Chemicals

Semax was purchased from Caslo (Kongens Lyngby, Denmark) with purity grade > 95%. aβ_1–16_, aβ_1–28_, and aβ_1–40_ were purchased from GenScript (Piscataway, NJ, USA) with purity grade > 95%. 3-CCA and BCA and all other salts were purchased from Sigma-Aldrich (St. Louis, MO, USA). All reagents and solvents were purchased and used as received unless otherwise noted.

### 4.2. aβ Peptide Preparation

Stock solutions of aβ_1–40_, aβ_1–16_, and aβ_1–28_ peptides were prepared by dissolving peptides (1 mg) in NH_4_OH (1 mL). These solutions were divided in aliquots (200 μL), frozen at −80°C, and then lyophilized overnight. The lyophilized samples were redissolved in 20 μL of 10 mM NaOH to prevent the formation of any preaggregated species and used immediately for each experiment.

### 4.3. UV Measurement of Ascorbate Consumption

A stock solution (20 mM) of ascorbate was prepared in Milli-Q water just before beginning the experiment and was used immediately. Cu(NO_3_)_2_ stock solution was prepared and standardized with ethylenediaminetetraacetic acid, as reported elsewhere [99]. Because of the instability of ascorbate solution, a new solution was prepared for each experiment. Ascorbate consumption was monitored by using a Jasco V-670 spectrophotometer. Intensity of ascorbate (150 μM) absorption band at *λ* = 265 nm was monitored as a function of time in 10 mM phosphate buffer, pH = 7.4, and after addition of 20 μM Cu(II), 25 μM of Semax, and/or 25 μM of aβ peptides into a 1 cm path-length quartz cell (slit width = 2 nm). Copper was the last in order of addition, to trigger the ascorbate oxidation and start the measurements. In all experiments, a 1:1:0.8 Semax/aβ/Cu(II) molar ratio was used. Results of all UV measurements are the average of three independent experiments.

### 4.4. UV-Vis Spectra of Ascorbate Consumption and Copper(I)-(BCA)_2_ Complex Formation

UV-Vis spectra of ascorbate (150 μM), BCA (52 μM), and a solution of both after adding of Cu(II) (16 μM) were recorded in 10 mM phosphate buffer, pH = 7.4, in the absence and in the presence of Semax 20 μM into a 1 cm path-length quartz cell (slit width = 2 nm).

### 4.5. Vis Spectra of Copper(II)-aβ and Copper(II)-Semax Complexes

Vis spectra of Cu(II)-aβ and Cu(II)-Semax complexes were recorded in 10 mM phosphate buffer, pH = 7.4, after addition of 20 μM Cu(II) in 25 μM of Semax and in 25 μM of aβ peptide solutions into a 1 cm path-length quartz cell (slit width = 2 nm). For the competition measurements, 25 μM of Semax was added to a 25 μM of Cu(II)-aβ and the resulting spectrum was recorded.

### 4.6. In Vitro Measurement of ROS Production

3-CCA was used to detect ROS formation; a stock solution (2 mM) was prepared in phosphate (10 mM) buffer at pH 9 at room temperature. The oxidation of nonfluorescent 3-CCA (20 μM) to the fluorescent 7-OH-CCA by OH^•^ was followed as an increase of fluorescence intensity over time at 450 nm upon excitation at 395 nm. The measurements were performed in a 1 cm path-length quartz cell by using a Horiba Fluoromax-4 fluorimeter. The excitation and emission slit widths were set at 2 nm. The samples for fluorescence assay were prepared using the same experimental conditions shown for UV measurements. Results are the average of three independent experiments.

### 4.7. Cell Culture

Human neuroblastoma cells (SH-SY5Y) were cultured in 5% CO_2_ under a humidified atmosphere at 37°C in an incubator (Heraeus Hera Cell 150 C incubator) in Dulbecco's modified eagle medium DMEM-F12 (Invitrogen) supplemented with 10% fetal bovine serum (FBS) (Invitrogen) and 1% penicillin/streptomycin (Invitrogen) in tissue culture–treated Corning® flasks (Sigma-Aldrich, St. Louis, MO, USA). Once cells reached 95% confluence, they were split (using 0.25% trypsin-EDTA; Invitrogen) into fractions and propagated or seeded to be used in experiments. Cell passage procedure (tripsinization) were performed weekly. Passages 10–30 were used for all the experiments.

### 4.8. Intracellular Detection of ROS by Flow Cytometry

Intracellular ROS were measured using H_2_DCFDA (MolecularProbes-Thermo Fisher Scientific), an oxidation-sensitive fluorescent probe. This test is based on the principle that endogenous intracellular esterases hydrolyze H_2_DCFDA, trapping free DCF inside cells. ROS, predominantly hydroperoxides, convert nonfluorescent H_2_DCFDA to the highly fluorescent DCF. Cells, seeded on 12 multiwell plate in 800 µL DMEM-F12 medium supplemented with 10% FBS, 1% penicillin/streptomycin of the day before, were rinsed with 800 µL of Dulbecco's phosphate-buffered saline (PBS) and loaded with 10 μM H_2_DCFDA for 30 min at 37°C in the dark. Cells were then washed twice with 800 µL of PBS and further incubated in 800 µL of PBS for an additional 60 min at 37°C with Cu(II)-ascorbate and Cu(II)-ascorbate-aβ in the presence or absence of Semax. H_2_O_2_ was used as a positive control. To avoid quenching phenomena due to the presence of serum (FBS) and/or phenol red in full media, all treatments were carried out in PBS. At the end of each treatment, cells were again washed with 800 µL of PBS before trypsinization, and the fluorescence was measured. 20,000 cells per sample were analyzed using a CyFlow® ML flow cytometer (Partec) system equipped with three laser sources and 10 optical parameters with dedicated filter setting and a high numerical aperture microscope objective (50× NA 0.82) for the detection of different scatter and fluorescence signals. The cells were excited by an air-cooled argon 488 nm laser and then the signal from DCF was read in FL1 log mode. Data obtained were acquired using the FlowMax software (Partec) and presented as histograms showing the percentage of DCF positive cells, based on the M1 region, calculated by using the FCS Express 5 Flow Research edition. M1 region was chosen considering the fluorescence intensity shift between untreated cells and treated cells.

### 4.9. Cell Viability

5000 cells per well in 200 μL of DMEM-F12 medium supplemented with 10% FBS, 1% penicillin/streptomycin, were seeded in 96 multiwell plate and were treated as described in the results section. Following 24 h of treatments, SH-SY5Y cell viability was estimated by using the MTT assay (3-(4,5-dimethylthiazol-2-yl)-2,5-diphenyltetrazolium bromide) as described previously [100]. MTT determination involves reduction of the tetrazolium salt MTT to purple formazan crystals in living cells. At the end of each treatment, 0.5 mg/mL MTT (Sigma) was added to the cells and incubated in Heraeus Hera Cell 150 C incubator at 37°C for 2 h. The formazan crystals produced by MTT reduction were dissolved by using 100 μL of DMSO (Sigma), and the absorbance (*λ* 569 nm) was measured by using the top reading mode of the Varioskan flash spectral scanning multimode microplate reader (Thermo Fisher Scientific), with a reference *λ* of 670 nm to subtract background.

### 4.10. Western Blotting

Western blotting analysis was performed on samples after different incubation times (0 and 30 min) at 37°C to assess any changes in the aggregation state of aβ (aβ 20 μM − Cu^2+^ 20 μM w/wo Semax 25 μM). For western blotting analysis, equal amounts of samples were loaded onto 4%–12% Bis-Tris gel (Invitrogen). After separation (160 V) using MES SDS Running buffer (Invitrogen), proteins were transferred by using Transfer buffer BOLT onto a nitrocellulose membrane, then the membrane was blocked with Intercept Blocking buffer (Invitrogen) and revealed by overnight incubation at 4°C, with the specific primary mouse monoclonal anti-aβ_1–16_ antibody (6E10, Covance) (dilution 1:1000). The appropriate infrared-dye labeled secondary goat anti-mouse IRDye 800-conjugated antibody (dilution 1:25,000, LI-COR Biosciences) was used to detect primary antibodies at RT for 45 min. Hybridization signals were detected by the means of the Odyssey Infrared Imaging System (LI-COR Biosciences).

### 4.11. Statistical Analysis

Results of biological assays were expressed as the mean ± SEM of at least three sets of independent experiments performed in triplicates and based on 5000 cell for each group (MTT) or 20000 (Flow Cytometry). One-way analysis of variance (ANOVA) followed by Tukey's post hoc test (MTT) (*p* < 0.05 was taken as significant (^∗^)) or by chi-square test (Flow Cytometry) (*p* < 0.001 was taken as significant (^∗^)) was used for comparisons between all groups.

## Data Availability

All data supporting the results are included within the article and in the Supporting Information.

## Nomenclature

ACTH(4–10): Sequence 4–7 of N-terminal domain of the adrenocorticotropic hormone
aβ: Amyloid-β peptide
aβ_1–16_: Amyloid-β peptide 1–16
aβ_1–28_: Amyloid-β peptide 1–28
aβ_1–40_: Amyloid-β peptide 1–40
3-CCA: Coumarin-3-carboxylic acid fluorescence assay
BCA: Bicinchoninic acid
H2DCFDA: Dichlorofluorescein diacetate
DCF: 2′,7′-dichlorofluorescein

## Supporting Information

Supporting Information includes: Supporting Figures, including representative western blot of 20 μM aβ_1–40_/20 μM Cu(II) upon 30 min of incubation in the presence or in the absence of 25 μM Semax; ascorbate consumption due to ROS formation and formation of OH^•^ measured by fluorescence of 7-OH-CCA in the presence of aβ_1–16,_ aβ_1–28,_ aβ_1-40_ and Cu^2+^; UV-Vis spectra of Semax + Cu^2+^, aβ + Cu^2+^, complex (Semax-Cu^2+^) + aβ and complex (aβ-Cu^2+^) + Semax; ascorbate consumption due to ROS formation and formation of OH^•^ measured by fluorescence of 7-OH-CCA in the presence of (aβ_1–16_ or aβ_1–28_ + Semax) + Cu^2+^ and in the presence of Semax added after 5 min at the preformed complex (aβ_1–16_ or aβ_1–28_ + Cu^2+^); determination of *β* for complexes involved in the equilibria (PDF).
