# Supplementary material for: Semax, a Copper Chelator Peptide, Decreases the Cu(II)-Catalyzed ROS Production and Cytotoxicity of aβ by Metal Ion Stripping and Redox Silencing
Source: Bioinorg Chem Appl. 2025 Jun 3;2025:4226220. doi: 10.1155/bca/4226220 (PMC12151629; doi:10.1155/bca/4226220)
Supplement: Supporting Information — Additional supporting information can be found online in the Supporting Information section. [file 4226220.f1.docx]

**Supporting Information**

**Semax, a copper chelator peptide, decreases the Cu(II) catalyzed ROS production and cytotoxicity of aβ by metal ion stripping and redox silencing.**

Marianna Flora Tomasello^1^, Maria Carmela Di Rosa^1^, Irina Naletova^1^, Michele Francesco Maria Sciacca^1^, Alessandro Giuffrida^2^, Giuseppe Maccarrone^2^, Francesco Attanasio^1^*.

* Corresponding author: [francesco.attanasio@cnr.it](mailto:francesco.attanasio@cnr.it)

^1^ Institute of Crystallography, CNR, P. Gaifami 18, 95126 Catania, Italy;

^2^ Department of Chemical Sciences, University of Catania, A. Doria 6, 95125 Catania, Italy;

**Table of Contents**

**Supporting Figures ……..**…………………………………………………………………………………..... **S3–7**

**Figure S1. Representative Western blot of 25 µM aβ_1-40_/20 µM Cu(II) upon 30 minutes of incubation in the presence or in the absence of 20 µM Semax** ……………………………………………….………..**S3**

**Figure S2.** Ascorbate consumption due to ROS formation and formation of OH^•^ measured by fluorescence of 7-OH-CCA formed in the presence of aβ_1-40_ and Cu^2+^, in the presence of aβ_1-28_ and Cu^2+^ and in the presence of aβ_1-16_ and Cu^2+^……………………….………………………………………………………………….……**S4**

**Figure S3.** UV-Vis spectra of Semax + Cu^2+^, aβ + Cu^2+^, complex (Semax-Cu^2+^) + aβ and complex (aβ- Cu^2+^) + Semax.………………………………………..………………………………………………………….....…**S5**

**Figure S4.** Ascorbate consumption due to ROS formation and formation of OH^•^ measured by fluorescence of 7-OH-CCA formed in the presence of (aβ_1-16_ + Semax) + Cu^2+^ and in the presence of Semax added after 5 minutes at the preformed complex (aβ_1-16_ +Cu^2+^) ………...……………………………………………..…**S6**

**Figure S5.** Ascorbate consumption due to ROS formation and formation of OH^•^ measured by fluorescence of 7-OH-CCA formed in the presence of (aβ_1-28_ + Semax) + Cu^2+^ and in the presence of Semax added after 5 minutes at the preformed complex (aβ_1-28_ +Cu^2+^).………………………………………………………….**S7**

**S6** Determination of β for complexes involved in the equilibria……………………….…..…………..... **S8–9**


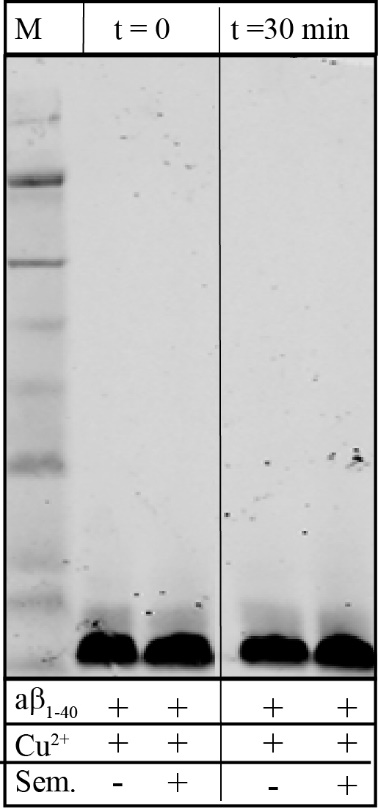


**Figure S1. Representative Western blot of 20 µM aβ_1-40_/20 µM Cu(II) upon 30 minutes of incubation in the presence or in the absence of 25 µM Semax.** Samples were separated onto a 4-12% bis-tris gel SDS-PAGE, and detected by using the monoclonal antibody 6E10 (recognizing residues 1-16 of aβ). The figure indicates that aβ_1-40_ does not form high molecular weight oligomeric species during the incubation period, regardless of the presence of Semax..1) aβ 20 µM, Cu^2+^ 20 µM t = 0 min; 2) aβ 20 µM, Cu^2+^ 20 µM, Semax 25 µM t = 0 min; 3) aβ 20 µM, Cu^2+^ 20 µM t = 30 min; 4) aβ 20 µM, Cu^2+^ 20 µM, Semax 25 µM t = 30 min.


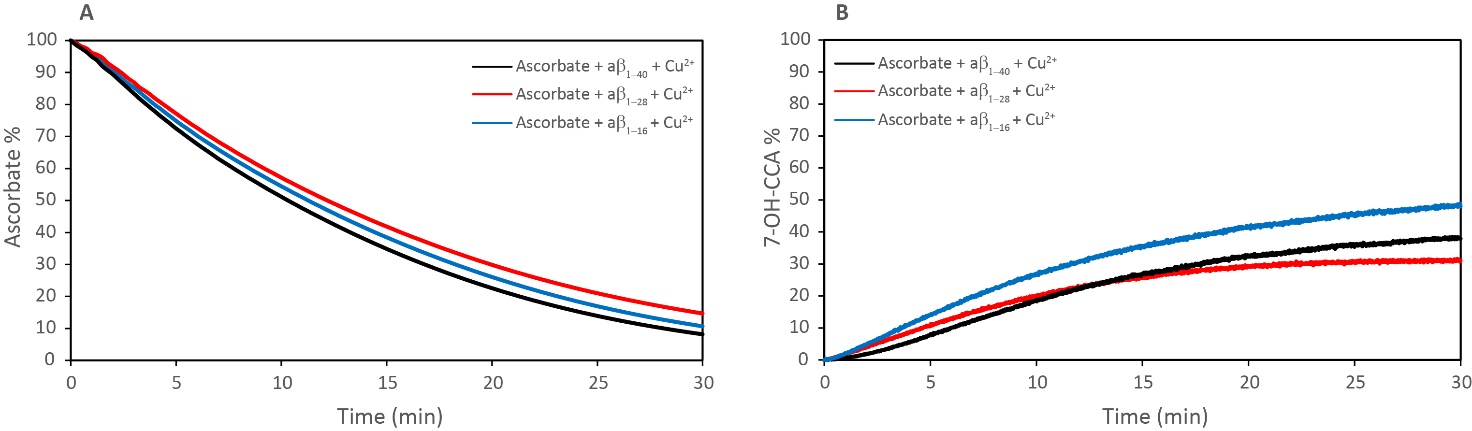


**Figure S2.** Panel A) Ascorbate consumption due to ROS formation in the presence of aβ_1-40_ and Cu^2+^ (black curve), in the presence of aβ_1-28_ and Cu^2+^ (red curve) and in the presence of aβ_1-16_ and Cu^2+^ (blue curve). Panel B) Formation of OH^•^ measured by fluorescence of 7-OH-CCA formed in the presence of aβ_1-40_ and Cu^2+^ (black curve), in the presence of aβ_1-28_ and Cu^2+^ (red curve) and in the presence of aβ_1-16_ and Cu^2+^ (blue curve). Concentration of Ascorbate was 150 µM. Concentrations of aβ_1-16_, aβ_1-28_, aβ_1-40_ and Cu2+ were 20 µM. All measurements were performed in 10 mM phosphate buffer, pH = 7.4.


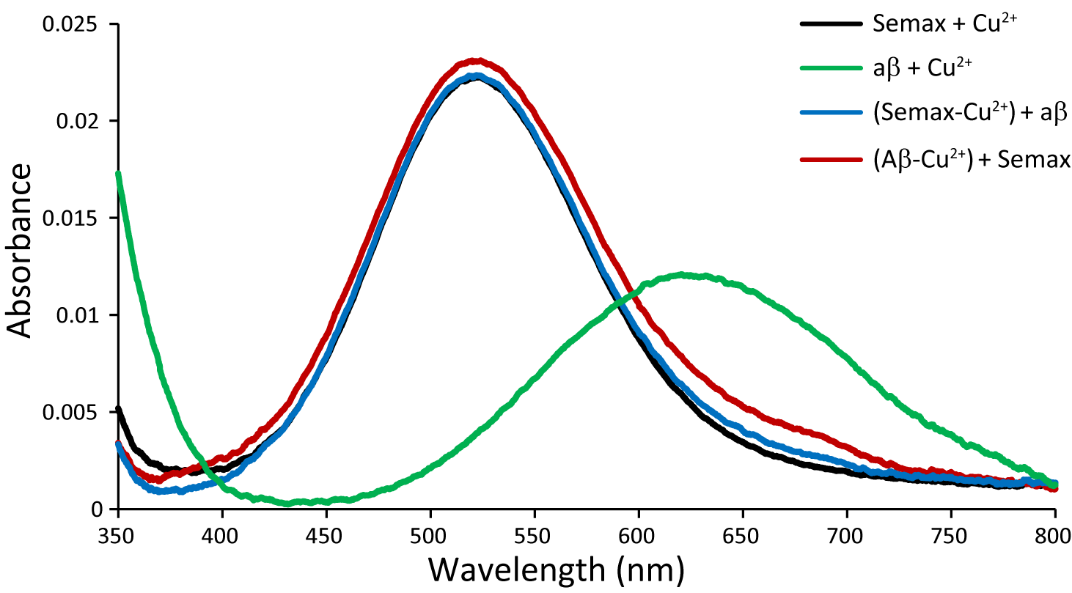


**Figure S3.** UV-Vis spectra of Semax + Cu^2+^ (black curve), aβ + Cu^2+^ (green curve), the preformed complex (Semax-Cu^2+^) + aβ (blue curve) and the preformed complex (aβ- Cu^2+^) + Semax (red curve). Concentrations of Semax, aβ and Cu^2+^ were 25, 20 and 20 µM respectively*.* All measurements were performed in 10 mM phosphate buffer, pH = 7.4.


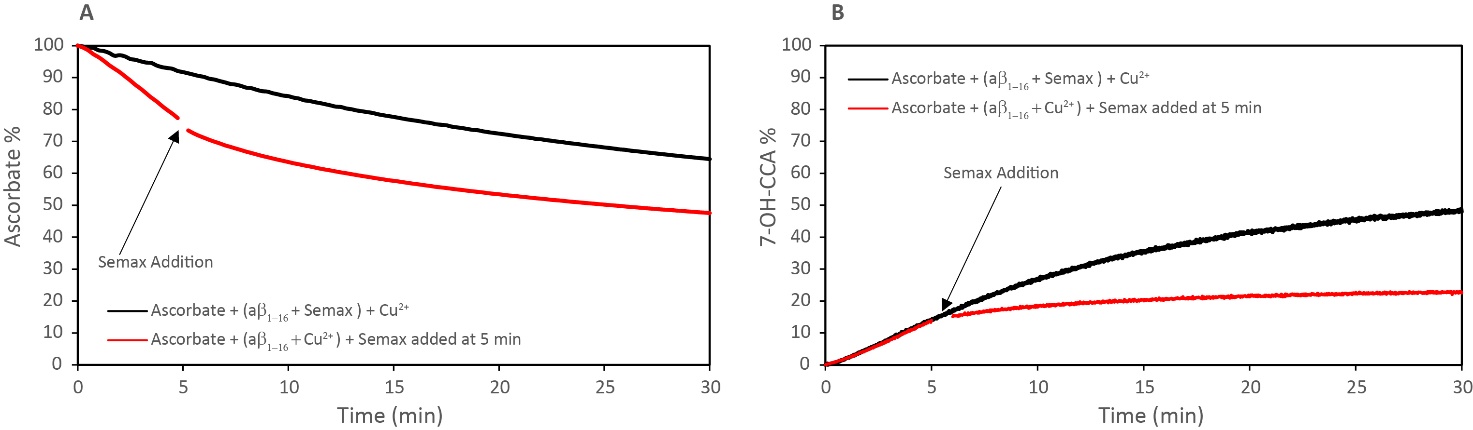


**Figure S4.** Panel A) Ascorbate consumption due to ROS formation in the presence of (aβ_1-16_ + Semax) + Cu^2+^ (black curve) and in the presence of Semax added after 5 minutes at the preformed complex (aβ_1-16_ +Cu^2+^) (red curve). Panel B) Formation of OH^•^ measured by fluorescence of 7-OH-CCA formed in the presence of (aβ_1-16_ + Semax) + Cu^2+^ (black curve) and in the presence of Semax added after 5 minutes at the preformed complex (aβ_1-16_ +Cu^2+^) (red curve). Concentration of Ascorbate was 150 µM. Concentrations of Semax, aβ_1-16_ and Cu^2+^ were 25, 20 and 20 µM respectively*.* All measurements were performed in 10 mM phosphate buffer, pH = 7.4.


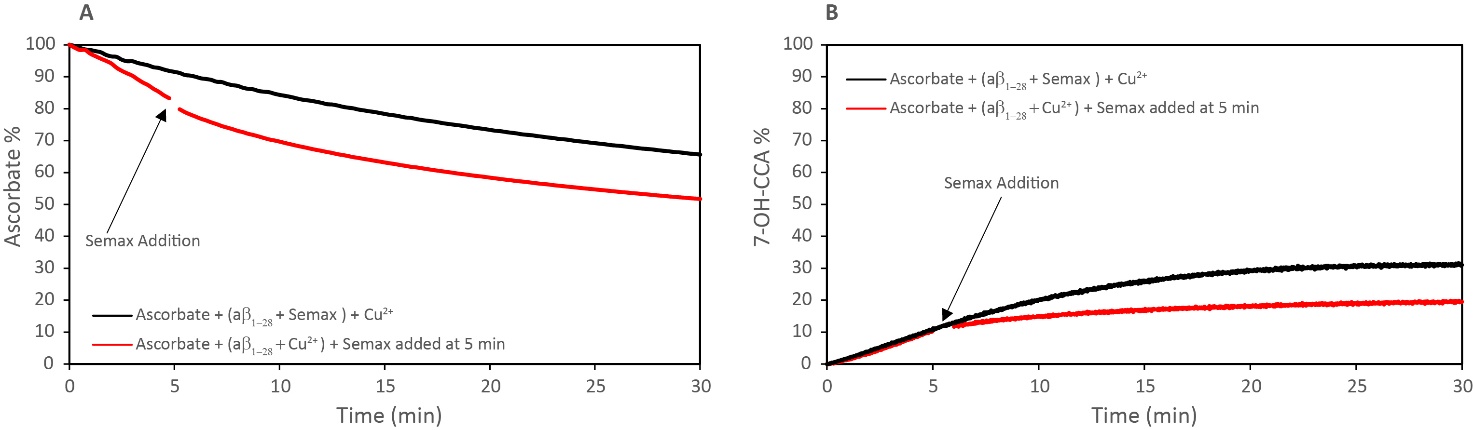


**Figure S5.** Panel A) Ascorbate consumption due to ROS formation in the presence of (aβ_1-28_ + Semax) + Cu^2+^ (black curve) and in the presence of Semax added after 5 minutes at the preformed complex (aβ_1-28_ +Cu^2+^) (red curve). Panel B) Formation of OH^•^ measured by fluorescence of 7-OH-CCA formed in the presence of (aβ_1-28_ + Semax) + Cu^2+^ (black curve) and in the presence of Semax added after 5 minutes at the preformed complex (aβ_1-28_ +Cu^2+^) (red curve). Concentration of Ascorbate was 150 µM. Concentrations of Semax, aβ_1-28_ and Cu^2+^ were 25, 20 and 20 µM respectively*.* All measurements were performed in 10 mM phosphate buffer, pH = 7.4.

***S6 Determination of β for complexes involved in the equilibria***

$$2{Cu}^{2+}+ASC \to2{Cu}^{+}+DHA$$

is characterized by the constant:

$$K= \frac{\left[ {Cu}^{+} \right]^{2} \left[ DHA \right]}{\left[ {Cu}^{2+} \right]^{2} \left[ ASC \right]}$$

those value can be calculated from the formal potential values for the two redox systems, i.e.:

$$E_{\frac{{Cu}^{2+}}{{Cu}^{+}}}^{f}=0.153 V E_{\frac{DHA}{ASC}}^{f}=0.055 V$$

$logK= \frac{2\left( 0.153-0.055 \right)}{0.059}$ $logK=3.32$

Actually, the reaction occurring in solution is:

$${Cu}^{2+}+2{BCA}^{2-}+ e^{-} \to{Cu\left( BCA \right)_{2}}^{3-}$$

$$E= E_{\frac{{Cu}^{2+}}{Cu\left( BCA \right)_{2}^{3-}}}^{f}+0.059\log\frac{\left[ {Cu}^{2+} \right] {\left[ {BCA}^{2-} \right]^{2}}}{\left[ Cu\left( BCA \right)_{2}^{3-} \right]}$$

those formal potential can be calculated by taking into account the formal potential for the couple Cu^2+^/Cu^+^:

$E= E_{\frac{{Cu}^{2+}}{{Cu}^{+}}}^{f}+0.059\log\frac{\left[ {Cu}^{2+} \right]}{\left[ {Cu}^{+} \right]}$ (1)

and from the following equilibrium and the corresponding stability constant:

${Cu}^{+}+2{BCA}^{2-}\to Cu\left( BCA \right)_{2}^{3-}$

$\beta=\frac{\left[ Cu\left( BCA \right)_{2}^{3-} \right]}{\left[ {Cu}^{+} \right] \left[ {BCA}^{2-} \right]^{2}}$ $log\beta=17.66$

$$\left[ Cu\left( BCA \right)_{2}^{3-} \right]= \beta\left[ {Cu}^{+} \right] \left[ {BCA}^{2-} \right]^{2}$$

$E= E_{\frac{{Cu}^{2+}}{Cu\left( BCA \right)_{2}^{3-}}}^{f}+0.059\log\frac{\left[ {Cu}^{2+} \right] {\left[ {BCA}^{2-} \right]^{2}}}{\beta\left[ {Cu}^{+} \right] \left[ {BCA}^{2-} \right]^{2}}$ (2)

By equalizing the members of equations (1) and (2) it was obtained that:

$$E_{\frac{{Cu}^{2+}}{Cu\left( BCA \right)_{2}^{3-}}}^{f}+0.059\log\frac{\left[ {Cu}^{2+} \right]}{\left[ {Cu}^{+} \right]}+0.059 log\frac{1}{\beta}= E_{\frac{{Cu}^{2+}}{{Cu}^{+}}}^{f}+0.059\log\frac{\left[ {Cu}^{2+} \right]}{\left[ {Cu}^{+} \right]}$$

$$E_{\frac{{Cu}^{2+}}{Cu\left( BCA \right)_{2}^{3-}}}^{f}= E_{\frac{{Cu}^{2+}}{{Cu}^{+}}}^{f}+0.059 log\beta$$

$$E_{\frac{{Cu}^{2+}}{Cu\left( BCA \right)_{2}^{3-}}}^{f}= 0.153+0.059 x 17.66$$

$$E_{\frac{{Cu}^{2+}}{Cu\left( BCA \right)_{2}^{3-}}}^{f}=1.195 V$$

This value clearly indicates that in the presence of BCA the system copper(II)/copper(I) is a more powerful oxidizing agent and thus it can be reduced more efficaciously by ASC.

Furthermore, from the two semi-reactions:

$${Cu}^{2+}+2{BCA}^{2-}+ e^{-} \to{Cu\left( BCA \right)_{2}}^{3-}$$

$$ASC \to DHA+2e^{-}$$

we obtain:

$${2Cu}^{2+}+4{BCA}^{2-}+ ASC \to{2Cu\left( BCA \right)_{2}}^{3-}+DHA$$

and the corresponding stability constant:

$$\beta= \frac{\left[ {Cu\left( BCA \right)_{2}}^{3-} \right]^{2} \left[ DHA \right]}{\left[ {Cu}^{2+} \right]^{2} \left[ {BCA}^{2-} \right]^{4}\left[ ASC \right]}$$

$$log \beta= \frac{2 x 1\left( E_{\frac{{Cu}^{2+}}{Cu\left( BCA \right)_{2}^{3-}}}^{f}-E_{\frac{DHA}{ASC}}^{f} \right)}{0.059}$$

$log\beta= \frac{2\left( 1.195 - 0.055 \right)}{0.059}$ $log\beta= 38.64$
